# Oral Administration of Branched-Chain Amino Acids Attenuates Atherosclerosis by Inhibiting the Inflammatory Response and Regulating the Gut Microbiota in ApoE-Deficient Mice

**DOI:** 10.3390/nu14235065

**Published:** 2022-11-28

**Authors:** Ziyun Li, Ranran Zhang, Hongna Mu, Wenduo Zhang, Jie Zeng, Hongxia Li, Siming Wang, Xianghui Zhao, Wenxiang Chen, Jun Dong, Ruiyue Yang

**Affiliations:** 1 The Key Laboratory of Geriatrics, Beijing Institute of Geriatrics, Institute of Geriatric Medicine, Chinese Academy of Medical Sciences, Beijing Hospital/National Center of Gerontology of National Health Commission, Beijing 100730, China; 2 Department of Cardiology, Beijing Hospital, National Center of Gerontology, Institute of Geriatric Medicine, Chinese Academy of Medical Sciences, Beijing 100730, China; 3 National Center for Clinical Laboratories, Institute of Geriatric Medicine, Chinese Academy of Medical Sciences, Beijing Hospital, National Center of Gerontology, Beijing 100730, China

**Keywords:** atherosclerosis, branched-chain amino acids, inflammation, gut microbiota, bile acids

## Abstract

Atherosclerosis (AS) is a chronic inflammatory disease that serves as a common pathogenic underpinning for various cardiovascular diseases. Although high circulating branched-chain amino acid (BCAA) levels may represent a risk factor for AS, it is unclear whether dietary BCAA supplementation causes elevated levels of circulating BCAAs and hence influences AS, and the related mechanisms are not well understood. Here, ApoE-deficient mice (ApoE^−/−^) were fed a diet supplemented with or without BCAAs to investigate the effects of BCAAs on AS and determine potential related mechanisms. In this study, compared with the high-fat diet (HFD), high-fat diet supplemented with BCAAs (HFB) reduced the atherosclerotic lesion area and caused a significant decrease in serum cholesterol (TC) and low-density lipoprotein cholesterol (LDL-C) levels. BCAA supplementation suppressed the systemic inflammatory response by reducing macrophage infiltration; lowering serum levels of inflammatory factors, including monocyte chemoattractant protein-1 (MCP-1), tumor necrosis factor-α (TNF-α), interleukin-1β (IL-1β) and interleukin-6 (IL-6); and suppressing inflammatory related signaling pathways. Furthermore, BCAA supplementation altered the gut bacterial beta diversity and composition, especially reducing harmful bacteria and increasing probiotic bacteria, along with increasing bile acid (BA) excretion. In addition, the levels of total BAs, primary BAs, 12α-hydroxylated bile acids (12α-OH BAs) and non-12α-hydroxylated bile acids (non-12α-OH BAs) in cecal and colonic contents were increased in the HFB group of mice compared with the HFD group. Overall, these data indicate that dietary BCAA supplementation can attenuate atherosclerosis induced by HFD in ApoE^−/−^ mice through improved dyslipidemia and inflammation, mechanisms involving the intestinal microbiota, and promotion of BA excretion.

## 1. Introduction

Atherosclerosis (AS) is a chronic inflammatory disease that narrows the arterial lumen through complex atherosclerotic plaque formation. AS is the common pathological basis of many cardiovascular and peripheral vascular diseases [1,2]. Cardiovascular disease (most commonly coronary atherosclerotic disease) is the leading cause of death in both developed and developing regions [3,4,5]. Early detection and early intervention represent effective strategies to reduce cardiovascular morbidity and mortality.

Atherosclerosis is often accompanied by dyslipidemia, including elevated serum cholesterol (TC), triglycerides (TGs), low-density lipoprotein cholesterol (LDL-C) levels and decreased high-density lipoprotein cholesterol (HDL-C) levels. Excess serum LDL-C deposition accumulates in the arterial wall endothelium and is oxidized to oxidized low-density lipoprotein (ox-LDL). Macrophages can recognize and engulf ox-LDL and convert it into foam cells, which accumulate to form streaks or lipid patches [1,6,7]. Atherosclerosis encompasses more than just LDL deposition in the artery wall; it is also a chronic inflammatory disease, and the inflammatory response is critical to AS progression. Endothelial damage, which frequently occurs during the early stages of AS, enhances the inflammatory response [8,9,10]. In addition, activated endothelial cells release intercellular cell adhesion molecule-1 (ICAM-1), vascular cell adhesion molecule-1 (VCAM-1) and other inflammatory factors, which help recruit monocytes. Numerous inflammatory molecules including interleukin (IL), chemokines, and tumor necrosis factor (TNF) are released throughout these processes, exacerbating inflammation [11]. Massive macrophages and inflammatory factors infiltrate the vessel wall in the later stages of AS. Similarly, macrophages release extracellular metalloproteases that hydrolyze extracellular matrix collagen fibers and increase plaque instability, increasing the risk of cardiovascular disease [12].

The diversity and homeostasis of the gut microbiota are closely related to the nutrition, metabolism, diseases, and other physiological processes of the host. Dysbiosis is closely associated with various metabolic diseases, such as obesity, metabolic-associated fatty liver disease and AS [13,14,15]. Several studies suggest that the gut microbiota plays an important role in the development of AS [16,17].

Bile acids (BAs) are catabolic metabolites of cholesterol and lipids, and their dysregulated synthesis and metabolism are associated with diseases, including insulin resistance, dyslipidemia, and AS [18,19,20]. Promoting BA synthesis can improve cholesterol metabolism, which may help to prevent and alleviate AS. Furthermore, BAs may affect host physiological activity and intestinal flora composition via the immune response and Farnesoid X receptor [20,21,22].

Many epidemiological studies have shown that elevated serum BCAA levels are independently associated with a high risk of coronary and cerebrovascular atherosclerotic disease [23,24,25,26]. However, animal studies have found that dietary BCAA supplementation significantly lowers TG levels in macrophages by decreasing very low-density lipoprotein (VLDL) uptake and subsequently inhibiting lipid accumulation and macrophage foam cell formation [27]. In addition, studies have found that gut bacteria can regulate BCAA biosynthesis, transport, and metabolism. Our previous study demonstrated that oral administration of BCAAs ameliorates high-fat diet-induced metabolic-associated fatty liver disease via gut microbiota-associated mechanisms [28]. Although high circulating BCAA levels may represent a risk factor for AS, it is unclear whether dietary BCAA supplementation causes elevated levels of circulating BCAAs and hence influences AS. It is also unknown whether dietary BCAA supplementation influences the development of AS through gut microbiota-related mechanisms. Therefore, the purpose of this study was to determine the effects of dietary BCAA supplementation on AS in ApoE^−/−^ mice and the underlying mechanisms, with a focus on gut microbiota remodeling and the inflammatory response.

## 2. Materials and Methods

### 2.1. Animal Studies

ApoE^−/−^ mice (9 weeks old, male) were purchased from Vital River Laboratory Animal Technology Co., Ltd. (Beijing, China). The mice were raised (2–3 mice per cage) in a special room with 22 °C, 40–70% humidity, and a 12-h light/dark cycle. After one week of adaptation to a normal diet, mice were randomly divided into the following four groups: normal chow diet group (ND, *n* = 10), normal chow diet supplemented with BCAAs group (NB, *n* = 10), high-fat diet group (HFD, *n* = 10), and high-fat diet supplemented with BCAAs group (HFB, *n* = 10). The normal chow diet (12% of kcal fat, 1025), normal chow diet supplemented with BCAAs, high-fat diet (41% of kcal fat with an extra supplement of 0.15% (*w*/*w*) cholesterol, H10141), and high-fat diet supplemented with BCAAs were purchased from Beijing HFK Bioscience Co., Ltd. (Beijing, China). Valine, L-leucine, and L-isoleucine were purchased from Nanjing Jingzhu Biotechnology Co., Ltd. (Nanjing, China). The extra amounts of L-leucine, L-isoleucine, and valine per 100 g diet supplemented with BCAAs were 0.56 g, 0.40 g, and 0.40 g, respectively. All mice had free access to food and water. Throughout the experiment, the padding and water were changed once a week, whereas the food of the HFD and HFB groups was changed twice a week to prevent sudor production via fat oxidation, which would subsequently affect model establishment. The amount of food consumed and the body weight were recorded every two weeks.

### 2.2. Sample Collection

After 12 weeks of feeding, the mice were anesthetized with pentobarbital (30 mg/kg) and sacrificed after blood samples were collected by cardiac puncture. The whole aorta and aortic root were separated for protein extraction and staining. The livers were collected and frozen immediately in liquid nitrogen and then stored at −80 °C. The contents of the cecum and colon were collected in a sterile manner, immediately frozen in liquid nitrogen, and then stored at −80 °C until use.

The animal experiments were approved by Peking University Biomedical Ethics Commitment Experimental Animal Ethics Branch (LA2022010) and complied with the Guide for the Care and Use of Laboratory Animals established by the National Institutes of Health (NIH).

### 2.3. Histological Analysis

The aortas were carefully obtained from the mice and stained with Oil Red O. The lesion area was calculated as a percentage of the total aorta area. The fresh aortic roots were collected and immediately fixed in 4% paraformaldehyde solution, dehydrated, embedded, and then sectioned. Paraffin-embedded sections (5 μm) were used for hematoxylin and eosin (HE) staining, Masson staining, and immunohistochemical staining; frozen sections (8 μm) were used for Oil Red O staining. Immunohistochemistry of aortic roots was performed with an anti-F4/80 antibody (Cell Signaling Technology, Inc., Beverly, MA, USA) and following the instructions provided.

### 2.4. Serum Biochemical Assays

Serum concentrations of total TC, TG, HDL-C, LDL-C, and glucose (Glu) were measured using a 7180 automatic biochemical analyzer (Hitachi Ltd., Tokyo, Japan), according to the manufacturer’s instructions. Wako Pure Chemical Industries, Ltd. reagents (Osaka, Japan) and their matching calibration and quality control were used to measure HDL-C by direct method, LDL-C by direct method, TG by deglycerin method, and TC by enzyme method, respectively. DiaSys Diagnostic Systems GmbH (Holzheim, Germany) reagents and their matching calibration and quality control were used to measure glucose by hexokinase method.

### 2.5. Assay of BCAAs in Serum

The previously published LC−MS/MS method was used to determine the concentrations of BCAAs (Val, Leu, Ile) in serum [29]. In brief, 50 µL of serum or standard solution was added to an equal volume of isotopically labeled internal standard solution; acetonitrile (containing 0.1% formic acid) was added for precipitation and extraction, and the supernatant was transferred to vials for LC−MS/MS detection. The separation was performed on an Agilent 1260 HPLC system equipped with a Kinetex HILIC column (2.6 µm, 2.1 mm × 150 mm), and mass spectrometry data were collected using an API 5500 system (AB Sciex, Framingham, MA, USA) in ESI positive ion mode and MRM mode.

### 2.6. Enzyme-Linked Immunosorbent Assay (ELISA)

ELISA analysis was used to determine serum interleukin-1β (IL-1β), tumor necrosis factor-α (TNF-α), monocyte chemoattractant protein-1 (MCP-1), and interleukin-6 (IL-6) levels according to the instructions provided by the kit. The mouse IL-1β ELISA kit, mouse TNF-α ELISA kit, and mouse MCP-1 ELISA kit were obtained from R&D Systems (Minneapolis, MN, USA), and the mouse IL-6 ELISA kit was purchased from Novus (St Charles, MO, USA).

### 2.7. Western Blotting

Protein expression was determined by Western blot assay. Protein lysate (Sigma-Aldrich, Taufkirchen, Germany) was used to extract total protein from aorta tissues and liver tissues, and total protein concentrations were determined using a BCA protein assay kit (Applygen Technologies, Beijing, China). A total of 30 micrograms of protein was separated in SDS−PAGE gels and transferred onto PVDF membranes (Roche, Indianapolis, IN, USA). After washing with TBST, the membranes were blocked with TBST (containing 5% skimmed milk) for 2 h at room temperature. In brief, membranes were incubated overnight with primary antibodies at 4 °C, washed with TBST, and incubated with secondary antibodies for 2 h at ambient temperature. The primary antibodies against phospho-AKT, AKT, phospho-NF-κB p65, NF-κB p65, phospho-BCKDH-E1α, BCKDH-E1α, and β-actin were purchased from Cell Signaling Technology Inc. (Beverly, MA, USA). Bands were visualized using an ECL detection kit (Applygen Technologies, Beijing, China). β-Actin served as an internal reference protein. Band intensity was assessed by densitometry and expressed as the mean density area using ImageJ software.

### 2.8. 16S rRNA Gene Sequencing

To determine cecal and colonic content, 16S ribosomal RNA (rRNA) gene sequencing was performed. Amplification of the high variant region 3 (V3) and the high variant region 4 (V4) of bacterial 16S rRNA was performed using the 338F primer (5′-ACTCCTACGGAGGGCAGCA-3′) and the 806R primer (5′-GGACTACHVGGGTWTCTAAT-3′) for Illumina deep sequencing. High-fidelity DNA polymerase was used for PCR amplification. The 16S gene sequencing procedure was performed using Beijing Biomarker Technologies Co., Ltd. (Beijing, China), and all results were based on sequencing reads and operational taxonomic units (OTUs).

### 2.9. Assessment of Bile Acids in Intestines

A UPLC−MS/MS method was used to assess sixteen bile acids, including cholic acid (CA), chenodeoxycholic acid (CDCA), deoxycholic acid (DCA), α-muricholic acid (α-MCA), β-muricholic acid (β-MCA), ursodeoxycholic acid (UDCA), hyodeoxycholic acid (HDCA), lithocholic acid (LCA), taurocholic acid (TCA), glycocholic acid (GCA), taurodeoxycholic acid (TDCA), glycodeoxycholic acid (GDCA), tauro-α-muricholic acid/tauro-β-muricholic acid (Tα-MCA/Tβ-MCA), tauroursodeoxycholic acid (TUDCA), glycoursodeoxycholic acid (GUDCA), taurohyodeoxycholic acid (THDCA) and taurolithocholic acid (TLCA), in cecal and colonic contents. After thawing at 4 °C for 30 min, the sample (~50 mg) was placed in a 1.5-mL centrifuge tube and weighed accurately. A total of 200 μL of water was added to a test tube, and the mixture was ultrasonicated for 5 min. Then, 1 mL of methanol was added. The sample was vortexed for 5 min followed by ultrasonic treatment for 1 h and centrifugation (4 °C, 12,000 rpm) for 5 min. The supernatant (100 μL) and isotope standard solutions (10 μL, 2 μg/mL CA-d4 and LCA-d4) were loaded into a 1.5-mL centrifuge tube and vortexed for 2 min. The mixture was then transferred to vials for LC−MS/MS testing. A Waters ACQUITY UPLC I-CLASS chromatograph (Waters, Milford, USA) equipped with a UPLC BEH Amide column (2.1 mm × 100 mm, 1.7 μm, Waters, USA) was used for separation. The column temperature was 50 °C. The mobile phase consisted of water/acetonitrile (10:1, *v*/*v*, containing 1 mmol/L ammonium acetate, phase A) and acetonitrile/isopropanol (1:1, *v*/*v*, phase B) with a flow rate of 0.26 mL/min. Analyte isolation was performed by elution gradient, and the injection volume was 5.0 μL. The MS data were collected using a QTRAP 6500+ LOW MASS system (AB Sciex, Framingham, MA, USA) in negative ion mode. The analysis was performed based on the following parameters: ion source voltage 2.0 kV, ion source temperature 150 °C, desolvation temperature 600 °C, desolvation gas flow 1000 L/h, cone voltage 21 V, cone gas flow 10 L/h.

Total bile acid concentrations were calculated as the sum of the concentrations of all sixteen bile acids. The primary BAs included CA, TCA, GCA, CDCA, α-MCA, β-MCA, and Tα-MCA/Tβ-MCA. The secondary BAs included DCA, GDCA, LCA, HDCA, UDCA, TLCA, THDCA, GUDCA, and TUDCA. 12α-Hydroxylated bile acids (12α-OH BAs) included CA, DCA, TCA, GCA, GDCA, and TDCA. Non-12α-hydroxylated bile acids (non-12α-OH BAs) included CDCA, α-MCA, β-MCA, LCA, HDCA, UDCA, Tα-MCA/Tβ-MCA, TLCA, THDCA, GUDCA, and TUDCA. The concentrations of primary BAs, secondary BAs, 12α-OH BAs, and non-12α-OH BAs were calculated based on the sum of the concentrations of each type of bile acid mentioned above.

### 2.10. Statistical Analysis

The experimental results are expressed as the mean ± standard error (SEM). The statistical software SPSS 26.0 (Armonk, NY, USA), GraphPad Prism 8.0 (San Diego, CA, USA), and R 3.6.1 (Vienna, Austria) were applied for data analysis, processing, and graph generation. Two-group comparisons were performed using a *t* test. Multiple group comparisons were made using ANOVA and Tukey’s post hoc test, and repeated measures data were statistically analyzed using multivariate analysis of variance (MANOVA). The alpha diversity indices, including ACE, Chao1, Simpson, and Shannon indices, and beta diversity indices, including principal coordinate analysis (PCoA), were evaluated by QI-IME. Data were analyzed and plotted using q-values ≤ 0.05 (obtained after *p* value correction) as a criterion for differential colony screening. Correlation analysis was performed using Spearman correlation analysis. A *p* value < 0.05 was considered to be statistically significant.

## 3. Results

### 3.1. BCAA Supplementation Reduces Atherosclerotic Plaques Induced by a High-Fat Diet in ApoE^−/−^ Mice

**Figure 1:**
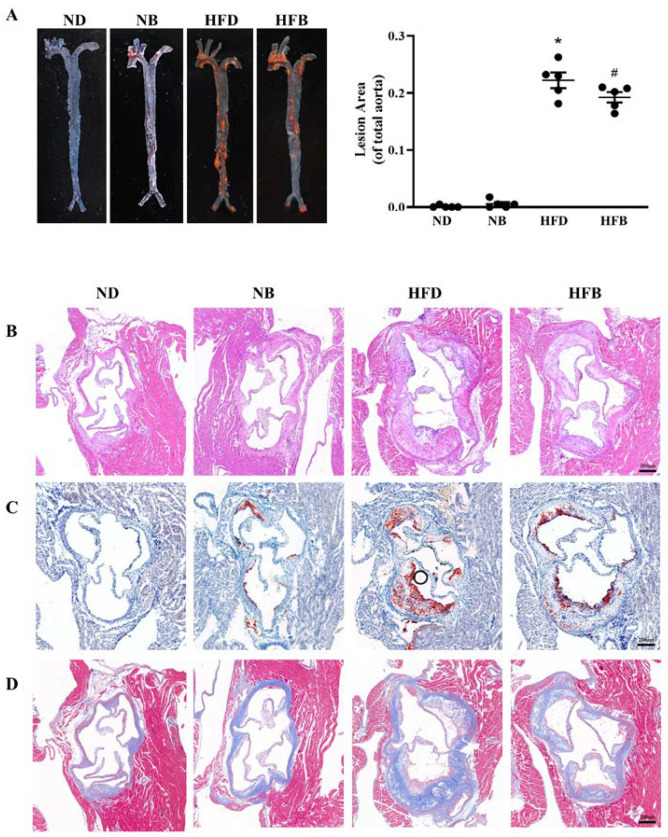
BCAA supplementation reduces atherosclerotic plaques induced by a high-fat diet in ApoE^−/−^ mice. (**A**) Representative images of Oil Red O staining of the aorta (left) and lesion area (right). The lesion area was expressed as a percentage of the total aorta area (*n* = 5 for each group). (**B**–**D**) Representative images of hematoxylin and eosin staining (**B**), Oil Red O staining (**C**), and Masson staining (**D**) of the aortic root (bar = 200 µm). The data are expressed as the mean ± standard error of the mean (SEM). * *p* < 0.05 vs. ND group; # *p* < 0.05 vs. HFD group.

AS is characterized by aortic plaque formation, and the lesion degree can be evaluated based on the size of atherosclerotic plaques. Therefore, the aorta was separated and stained with Oil Red O after feeding ApoE^−/−^ mice for 12 weeks to assess the atherosclerotic plaque, and the results are displayed in Figure 1A. The plaque area of whole aorta in the NB group was slightly increased compared with that in the ND group, but the difference was not statistically significant. The plaque area of whole aorta in the HFD group was significantly larger than that in the ND group, whereas the plaque area in the HFB group was significantly smaller than that in the HFD group. The aortic root atherosclerotic plaque area is also commonly utilized to assess atherosclerotic lesions. As shown in Figure 1B,C, the plaque area of the aortic root in the HFD group was greatly enlarged compared with that in the ND group, and the plaque area in the HFB group was markedly less than that in the HFD group. The aorta root intima in the ND group was smooth with neatly arranged endothelial cells and no atherosclerotic plaque formation, whereas very few atherosclerotic plaques were noted in the NB group. In the HFD group, apparent plaque lesions were noted with thickening aortic intima, an increased number of foam-like cells, and willow-shaped crystals inside the plaque. Compared with the HFD group, the plaque area and number of foam-like cells were remarkably decreased in the HFB group. These results suggest that dietary BCAA supplementation reduced atherosclerotic plaques induced by a high-fat diet in ApoE^−/−^ mice. Plaque stability was examined in this research by Masson staining of aortic roots. In Figure 1D, the collagen fiber content was apparently lower in the HFB group than in the HFD group.

### 3.2. BCAA Supplementation Ameliorates Dyslipidemia Induced by a High-Fat Diet in ApoE^−/−^ Mice

**Figure 2:**
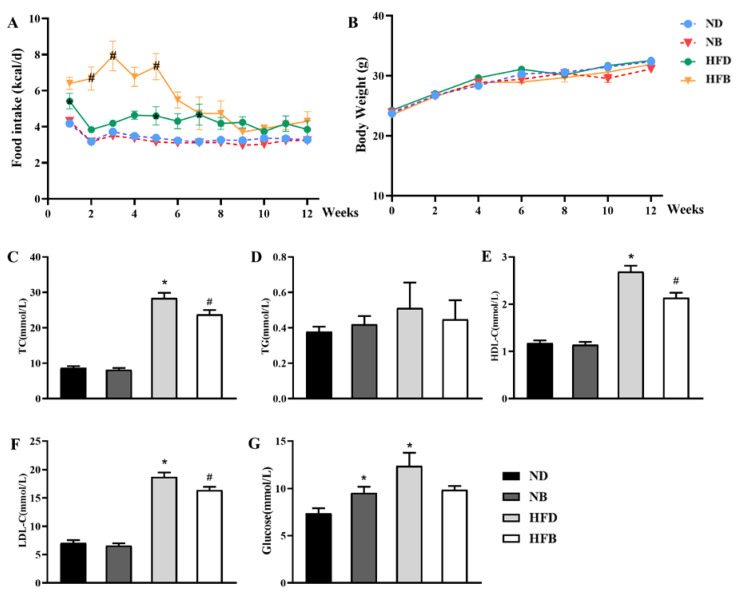
BCAA supplementation ameliorates dyslipidemia induced by a high-fat diet in ApoE^−/−^ mice. (**A**,**B**) The food intake (**A**) and body weight growth curve (**B**) of ApoE^−/−^ mice (0–12 weeks). The data were analyzed by two-way ANOVA. (**C**–**G**) Serum concentrations of (**C**) TC, (**D**) TG, (**E**) HDL-C, (**F**) LDL-C, and (**G**) glucose. The data are expressed as the mean ± SEM (*n* = 8 for each group). * *p* < 0.05 vs. ND group; # *p* < 0.05 vs. HFD group.

The difference in food intake between the ND and NB groups was not significant. The HFD group exhibited greater food intake compared with that of the ND group, and HFB group mice had significantly higher food intake compared with HFD group mice over the first 5 weeks (Figure 2A). However, neither a high-fat diet nor BCAA supplementation resulted in significant changes in body weight (Figure 2B).

As shown in Figure 2C–F, serum TC, LDL-C, and HDL-C levels were significantly increased in the HFD group compared with the ND group. The levels in the HFB group were significantly reduced compared with those in the HFD group. TC, TG, HDL-C, and LDL-C levels did not significantly differ between the NB group and the ND group. This finding suggests that dietary supplementation with BCAAs improves dyslipidemia induced by a high-fat diet in ApoE^−/−^ mice. Additionally, the blood glucose levels of the NB and HFD groups were significantly higher than those in the ND group. Compared with the HFD group, the blood glucose levels in the HFB group decreased, but the difference was not significant (Figure 2G).

### 3.3. Effects of BCAA Supplementation on Serum Levels of BCAAs and BCAA Metabolic Enzymes in ApoE^−/−^ Mice

**Figure 3:**
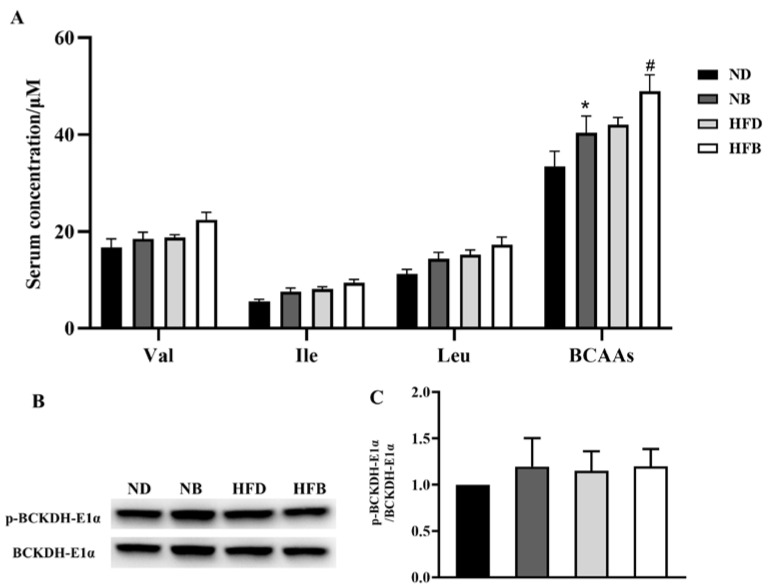
Effects of BCAA supplementation on serum levels of BCAAs and BCAA metabolic enzymes in ApoE^−/−^ mice. (**A**) Serum Val, Ile, Leu and total BCAA concentrations were determined by an LC−MS/MS method. (**B**) Western blotting was used to assess *p*-BCKDH-E1α and BCKDH-E1α protein expression in the liver. (**C**) The relative quantitative values for *p*-BCKDH-E1α/BCKDH-E1α are indicated. Densitometry values were normalized to β-actin. The data are expressed as the mean ± SEM (*n* = 8 for each group). * *p* < 0.05 vs. ND group; # *p* < 0.05 vs. HFD group.

As shown in Figure 3A, compared with the ND group, the total levels of the three BCAAs were elevated in the NB group, but the concentrations of Val, Ile, and Leu did not significantly differ between these two groups. Similarly, only the total levels of three BCAAs increased in the HFB group compared with the HFD group. BCAAs cannot be synthesized in mice and must be obtained through dietary supplementation. However, BCAAs can be catabolized in vivo, so BCAA circulating levels are also affected by BCAA catabolism. BCKDH is the rate-limiting enzyme in BCAA catabolism, and its activity is dependent on the level of phosphorylation of its E1α subunit. The expression levels of hepatic *p*-BCKDH-E1α and BCKDH-E1α were examined using Western blotting. The *p*-BCKDH-E1α/BCKDH-E1α ratio did not differ significantly among the four groups (Figure 3B,C).

### 3.4. BCAA Supplementation Relieves Inflammation Induced by a High-Fat Diet in ApoE^−/−^ Mice

**Figure 4:**
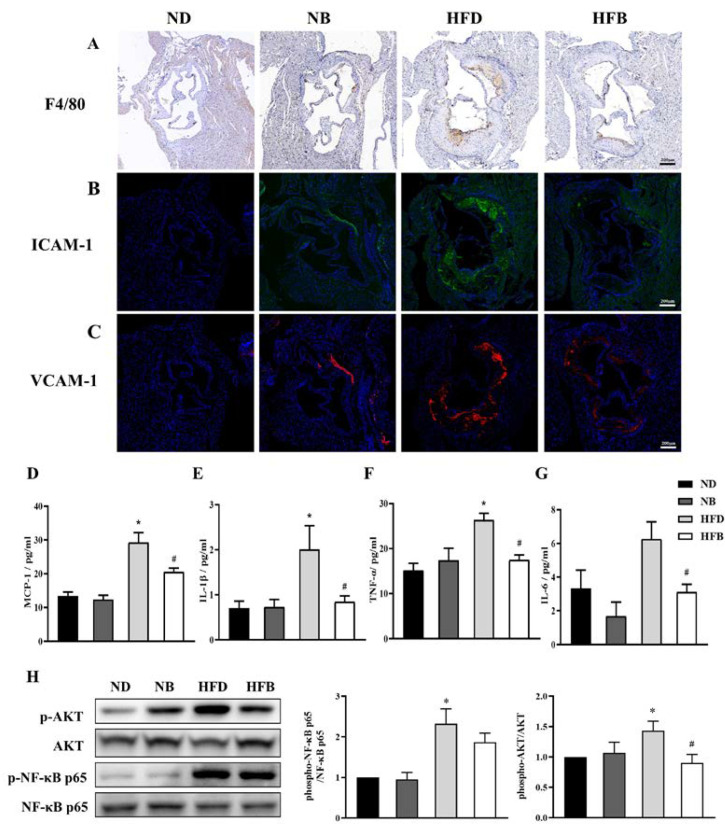
BCAA supplementation relieves inflammation induced by a high-fat diet in ApoE^−/−^ mice. (**A**) Representative F4/80 immunohistochemical sections of aortic root (bar = 200 µm). (**B**,**C**) ICAM-1 (**B**) and VCAM-1 (**C**) expression at the aortic root was determined by immunofluorescence staining. (**D**–**G**) The expression of inflammatory cytokines, including MCP-1 (**D**), IL-1β (**E**), TNF-α (**F**), and IL-6 (**G**), was measured using ELISA kits. (**H**) Representative western blot bands of AKT, *p*-AKT, NF-κB, and *p*-NF-κB in the aortic root (left). The relative quantitative data for AKT/*p*-AKT (middle) and NF-κB/*p*-NF-κB (right) are indicated. The data are expressed as the mean ± SEM (*n* = 8 for each group). * *p* < 0.05 vs. ND group; # *p* < 0.05 vs. HFD group.

F4/80 immunohistochemistry staining was applied to assess macrophage infiltration in aortic roots (Figure 4A). Macrophage infiltration was increased in the NB and HFD groups compared with the ND group, and was decreased in the HFB group compared with the HFD group. Immunofluorescence staining was performed on aortic root sections to examine the expression of intercellular cell adhesion molecule-1 (ICAM-1) and vascular cell adhesion molecule-1 (VCAM-1), which are important indicators of the inflammatory response (Figure 4B,C). Compared with the ND group, ICAM-1 and VCAM-1 expression was increased at the aortic root in the NB group and markedly elevated in the HFD group. These levels were dramatically downregulated in the HFB group compared with the HFD group.

In addition, as shown in Figure 4D–G, the levels ofMCP-1, IL-1β, and TNF-αwere significantly increased in the HFD group compared with the ND group. In addition, the levels of IL-6 were increased, but the result was not statistically significant. MCP-1, IL-1β, TNF-α, and IL-6 levels were significantly decreased in the HFB group compared with the HFD group.

The expression levels of protein kinase B (AKT) and nuclear factor κB (NF-κB), the upstream regulatory molecules of inflammatory factors, were further analyzed, and the data are provided in Figure 4H. The *p*-AKT/AKT ratio was significantly higher in the HFD group than in the ND group. The *p*-AKT/AKT ratio was significantly lower in the HFB group than in the HFD group. The *p*-NF-κB/NF-κB ratio in the NB group did not differ significantly from that in the ND group, whereas it increased significantly in the HFD group. The *p*-NF-κB/NF-κB ratio in the HFB group tended to be lower than that in the HFD group, but the difference was not statistically significant. These results suggest that dietary BCAA supplementation could alleviate the inflammatory response induced by a high-fat diet by potentially affecting the NF-κB/AKT pathway.

### 3.5. BCAA Supplementation Alters Intestinal Flora Diversity in ApoE^−/−^ Mice

**Figure 5:**
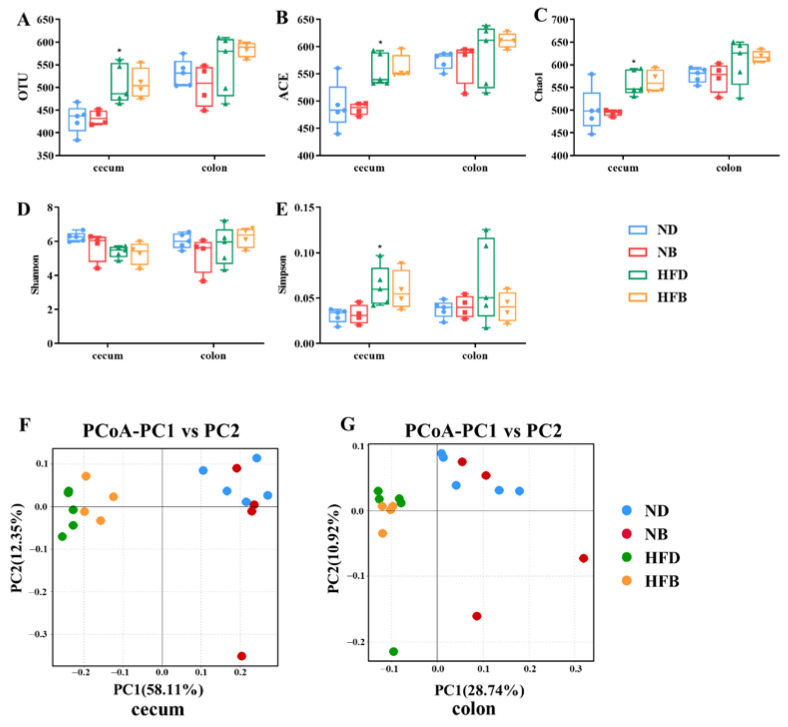
BCAA supplementation alters gut microbiota diversity in ApoE ^−/−^ mice. (**A**–**E**) Alpha diversity of gut microbiota in the cecum and colon was assessed by OTU (**A**), ACE index (**B**), Chao1 index (**C**), Shannon index (**D**), Simpson index (**E**). The data are presented as the mean ± SEM (*n* = 4–5 for each group). (**F**) PCoA based on UniFrac distance of gut microbiota in the cecum (**F**) and colon (**G**), *n* = 4−5 for each group. * *p* < 0.05 vs. ND group.

Here, 16S rRNA gene sequencing was performed to examine and analyze the intestinal bacteria in the cecum and colon, respectively, to investigate the effect of dietary supplementation with BCAAs on the diversity of intestinal flora in ApoE^−/−^ mice (Figure 5). Microbial diversity can be measured by two parameters: alpha diversity and beta diversity. Alpha diversity is generally applied to reveal the abundance and diversity of gut microbiota, with indicators including OTU, ACE, Chao1, Shannon indexes, and Simpson indexes. The alpha-diversity analysis (Figure 5A–E) shows that a high-fat diet increased the richness and diversity of intestinal flora, whereas BCAA supplementation had no significant effect on the alpha diversity of intestinal flora. Beta diversity analysis was employed to analyze the overall structural changes in the gut microbiota. PCoA based on UniFrac distance revealed distinct and separate clustering of microbiota compositions between HFD mice and ND mice, and between HFD mice and HFD supplemented with BCAA, and between ND mice and ND supplemented with BCAA. (Figure 5F,G). Overall, the beta diversity of intestinal flora in the cecum and colon of ApoE^−/−^ mice was changed by BCAA supplementation in both the normal and high-fat diets, and this alteration was more obvious when mice were fed a high-fat diet.

### 3.6. BCAA Supplementation Alters Gut Microbiota Composition in ApoE^−/−^ Mice

**Figure 6:**
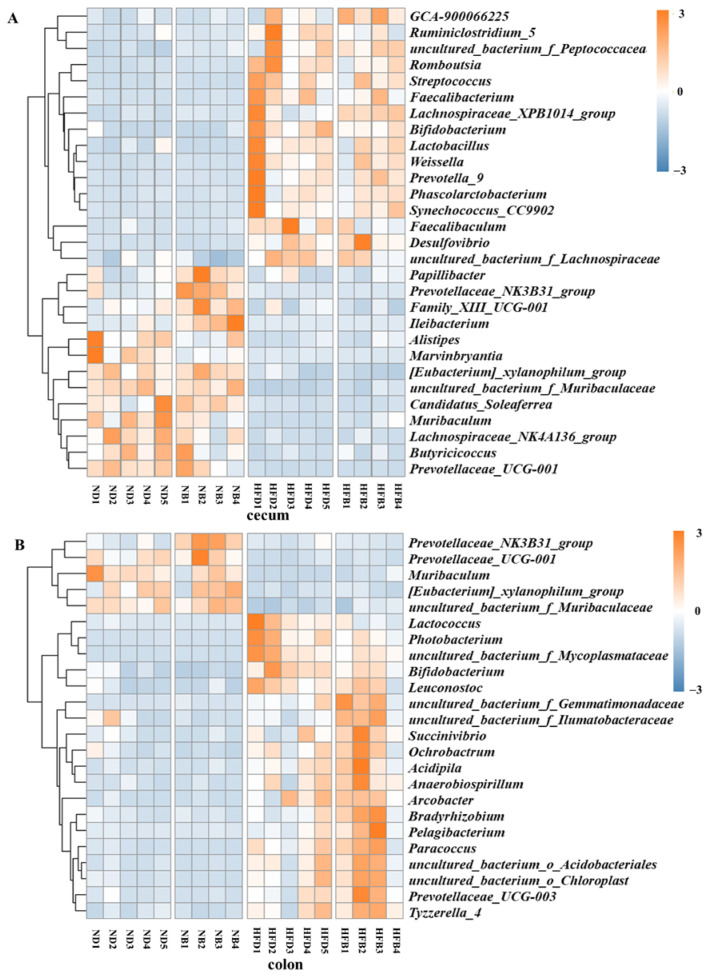
BCAA supplementation alters gut microbiota composition in ApoE^−/−^ mice. Heatmap of gut microbiota composition at the genus level for each group in the cecum (**A**) and colon (**B**), *n* = 4–5 per group. The scale bar indicates the standardized *Z* value of the microbial relative abundance. Red and blue colors indicate higher and lower average expression, respectively.

After ApoE^−/−^ mice were fed for 12 weeks, the effects of dietary supplementation with BCAAs on gut microbiota composition were investigated. The results of the heatmap and clustering analysis based on the relative abundance of intestinal flora are shown in Figure 6. The graphs were based on the relative abundance of bacteria in the cecum, as shown in Figure 6A. Compared with the ND group, the relative abundances of *Papillibacter*, *Prevotellaceae_NK3B31_group*, *Family_XIII_UCG-001* and *Ileibacterium* were upregulated, whereas the relative abundances of *Alistipes* and *Marvinbryantia* were downregulated in the NB group. Compared with the ND group, the relative abundances of *GCA-900066225*, *Ruminiclostridium_5* and *uncultured_bacterium_f_Peptococcaceae* at the genus level were significantly upregulated in the HFD group, and the relative abundances of *Papillibacter*, *Prevotellaceae*_*NK3B31*_*group* and *Family*_*XIII*_*UCG*-*001* were significantly downregulated at the genus level. The relative abundances of *Ruminiclostridium*_*5*, *Faecalibaculum* and *uncultured_bacterium_f_ Lachnospiraceae* were upregulated in the HFB group compared to the HFD group.

Heatmaps based on the relative abundance of colonic bacteria in mice are presented in Figure 6B. The relative abundances of the 24 different bacterial groups at the genus level revealed differences in the abundance and composition of the intestinal flora in each group. The relative abundance of *Prevotellaceae_NK3B31_group* was significantly higher in the NB group than in the ND group. Compared to the ND group, the relative abundances of *Prevotellaceae_UCG-001, Muribaculum*, [*Eubacterium]_xylanophilum_group* and *uncultured_bacterium_f_Muribaculaceae* were significantly lower, whereas *Lactococcus*, *Photobacterium*, *uncultured_bacterium_f_Mycoplasmataceae*, *Bifidobacterium* and *Leuconostoc* had significantly higher relative abundances in the HFD group. In addition, 10 genus levels of *Succinivibrio*, *Ochrobactrum*, and *Acidipila* also exhibited elevated intestinal flora abundances in the HFD group. Compared with the HFD group, the relative abundance of *Lactococcus*, *Photobacterium*, *uncultured_bacterium_f_Mycoplasmataceae* and *Bifidobacterium* decreased in the HFB group, and that of *uncultured_bacterium_f_ Gemmatimonadaceae*, *uncultured_bacterium_f_Ilumatobacteraceae* and *Succinivibrio* showed significantly higher levels of 14 genera in the intestinal flora.

**Figure 7:**
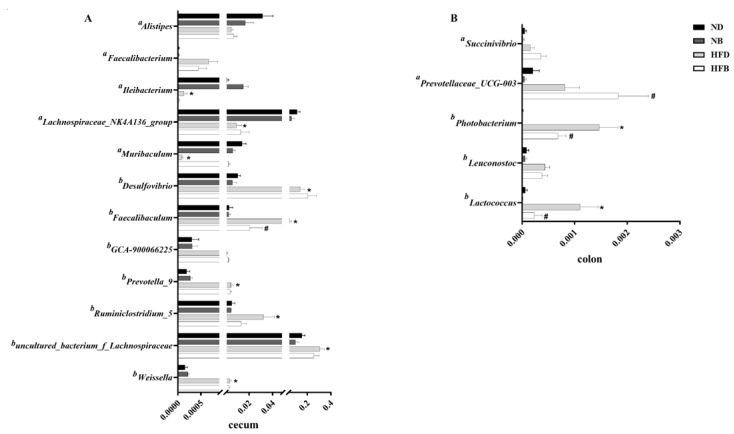
BCAA supplementation alters gut microbiota relative abundance in ApoE^−/−^ mice. The relative abundance of gut microbiota at the genus level in the cecum (**A**) and colon (**B**) The data are presented as the mean ± SEM (*n* = 4–5 for each group). Letters “a” and “b” indicate possible probiotics and pathogenic bacteria previously reported, respectively. * *p* < 0.05 vs. ND group; # *p* < 0.05 vs. HFD group.

To further determine the changes in flora among different groups, the flora with significantly altered relative abundances were analyzed, and the statistical results are shown in Figure 7. In the cecum, the relative abundances of *Desulfovibrio*, *Faecalibaculum*, *Prevotella*_*9*, *Ruminiclostridium*_*5*, *uncultured*_*bacterium*_*f_Lachnospiraceae* and *Weissella* were significantly higher in the HFD group compared with the ND group, whereas the relative abundances of *Ileibacterium*, *Lachnospiraceae_NK4A136*_*group* and *Muribaculum* were significantly lower than that in the ND group. The relative abundance of *Desulfovibrio* in the HFB group was significantly higher than that in the HFD group, whereas the relative abundance of *Faecalibaculum* was significantly lower than that in the HFD group. In addition, the relative abundances of *Lactococcus* and *Photobacterium* in the HFD group in the colon were significantly higher than that in the ND group, and the relative abundances noted in the HFB group were significantly lower than that in the HFD group. The relative abundance of *Prevotellaceae_UCG-003* in the HFB group was significantly higher than that in the HFD group.

### 3.7. BCAA Supplementation Promotes Bile Acid Excretion in ApoE^−/−^ Mice

**Figure 8:**
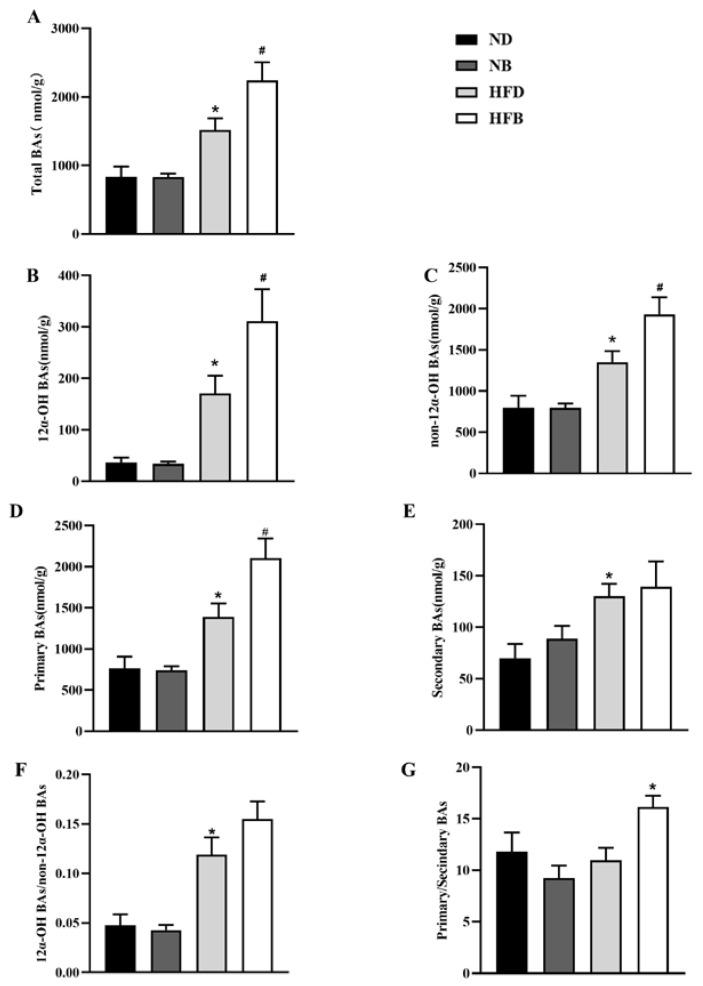
BCAA supplementation alters the bile acid composition in intestinal contents in ApoE^−/−^ mice. Sixteen molecular species of BAs were determined in cecal and colonic contents by an LC−MS/MS method. (**A**) Total bile acid concentrations. (**B**) 12α-OH BA concentrations. (**C**) Non-12α-OH BA concentrations. (**D**) Primary bile acid concentrations. (**E**) Secondary bile acid concentrations. (**F**,**G**) The ratios of 12α-OH BAs to non-12α-OH BAs (**F**) and ratios of primary BAs to secondary BAs (**G**). The data are presented as the mean ± SEM (*n* = 7–8 for each group). * *p* < 0.05 vs. ND group; # *p* < 0.05 vs. HFD group.

Dyslipidemia is an important risk factor for AS, and cholesterol can be metabolized to BAs in the liver, representing the main pathway of cholesterol metabolism. The concentrations of 16 types of BAs in the intestinal contents were assayed using LC−MS/MS. As shown in Figure 8, the levels of total BAs, 12α-OH BAs, non-12α-OH BAs, primary BAs, and secondary BAs and the ratio of 12α-OH/non-12α-OH BAs were significantly increased in the HFD group compared with the ND group. We also found that BCAA supplementation in the high-fat diet markedly increased the levels of total BAs, 12α-OH BAs, non-12α-OH BAs, primary BAs, and the ratio of primary/secondary BAs in the intestinal contents, but no significant differences in secondary BAs and the ratio of 12α-OH/non-12α-OH BAs were observed between the HFB and HFD groups. These results suggest that dietary supplementation with BCAAs may facilitate cholesterol catabolism by promoting BA excretion. 

### 3.8. Correlation of Intestinal Flora with Blood Glucose, Lipid Indices and Bile Acids

**Figure 9:**
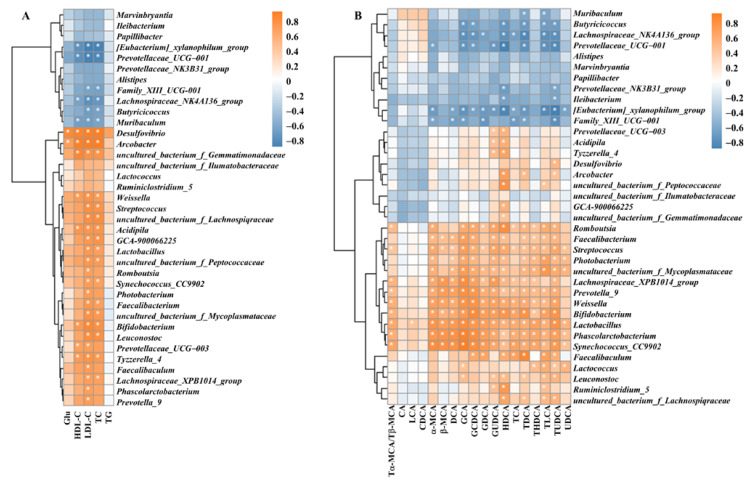
Spearman rank correlation analysis. (**A**) Spearman rank correlation analyses between intestinal microbiota and serum biochemical parameters, *n* = 4–5 per group. (**B**) Spearman rank correlation analyses between intestinal microbiota and bile acids in the cecum and colon, *n* = 4–5 per group. Red indicates positive correlation coefficients, and blue indicates negative correlation coefficients. Significant correlations are indicated by * *p* < 0.05.

The above results showed that dietary supplementation with BCAAs altered lipid profiles, BA levels, and intestinal flora diversity in ApoE^−/−^ mice; thus, the correlation of intestinal flora with blood glucose, lipid indices, and BA levels in mice was further analyzed. Spearman correlation analysis (Figure 9) revealed that the relative abundances of *Eubacterium xylanophilum group*, *Prevotellaceae*, *Lachnospiraceae NK4A136 group*, and *Muribaculum* were significantly negatively correlated with HDL-C, LDL-C, and TC levels. The relative abundances of *Desulfovibrio* and *Arcobacter* were significantly and positively correlated with GLU, HDL, LDL, and TC levels. The relative abundance of the intestinal flora of 5 genera, including *uncultured_bacterium_f_Gemmatimonadaceae,* was positively correlated with HDL-C, LDL-C, and TC levels. The relative abundance of intestinal flora of 10 genera, including *Streptococcus,* was significantly and positively correlated with LDL and TC levels. The relative abundance of *Prevotellaceae_UCG-003* was significantly and positively correlated with HDL-C and LDL-C levels. In addition, the heatmap correlation analysis (Figure 9B) showed that the relative abundance of intestinal flora of 8 genera, including *Muribaculum*, was significantly and negatively correlated with the levels of multiple BAs, while the relative abundance of intestinal flora of 17 genera, including *Romboutsia*, was significantly positively correlated with the levels of several BAs.

## 4. Discussion

AS is a common pathophysiological foundation for several types of cardiovascular and cerebrovascular diseases that involves complicated interactions among various factors. The pathogenesis of AS is sophisticated but not completely elucidated. In the present study, we investigated the effect of BCAA supplementation on AS and its mechanism in ApoE^−/−^ mice fed with a high-fat diet. BCAA supplementation substantially reduced the formation of aortic plaques, ameliorated dyslipidemia by enhancing the excretion of BAs, and alleviated the inflammatory response by potentially affecting the inflammatory related pathway. In addition, BCAA supplementation altered the gut bacterial beta diversity and gut microbiota composition and abundance, especially reducing harmful bacterial abundance and increasing probiotic abundance.

Atherosclerotic plaque formation is the most significant lesion characteristic of AS, and the plaque area and size directly indicate the extent of atherosclerotic lesions. In this study, high-fat feeding ApoE^−/−^ mice had massive plaques on the interior of the aorta, suggesting that the AS model had been successfully established. The lesion area dramatically decreased in the HFB group, indicating that BCAA dietary supplementation could attenuate AS induced by a high-fat diet. Furthermore, HE and Oil Red O-stained aortic root slices revealed that BCAA supplementation reduced the atherosclerotic plaque area. Zhao et al. also found that adding leucine to drinking water also significantly reduced the size of aortic atherosclerotic lesions in ApoE^−/−^ mice [30]. Plaque stability is another important indicator when evaluating the risk of plaque formation. It has been demonstrated that an elevated collagen fiber content plays an important role in enhancing plaque stability [31]. Masson staining results from this study revealed a decrease in collagen content in the HFB group compared to the HFD group. Considering that except for collagen fibers, plaque stability is also affected by factors such as lipids, macrophages, and smooth muscle cells [32,33,34], further experiments are required to verify the effect of BCAAs on plaque stability.

Dyslipidemia is widely recognized as an important risk factor for AS. In vivo, LDL-C is the vehicle for cholesterol metabolic transport, and excessive LDL-C deposition can cause subintimal migration and aggregation, damaging the vascular endothelium, increasing the number of foam cells, and ultimately resulting in AS [35,36]. A cross-sectional study showed that plasma BCAA levels are positively correlated with LDL-C levels, and increased BCAA levels are also positively associated with metabolic dyslipidemia [37]. Oren Rom et al. demonstrated that leucine reducedTG content in macrophages by inhibiting VLDL uptake in vitro, thereby inhibiting lipid accumulation and macrophage foam cell formation [38]. Zhao et al. demonstrated that adding BCAAs (leucine) to drinking water improved the lipid profile by promoting ATP-binding cassette transporter G5 (ABCG5)/ABCG 8-mediated hepatic cholesterol efflux in ApoE^−/−^ mice [30]. Similarly, in this study we revealed that dietary BCAA supplementation alleviates high-fat diet-induced dyslipidemia by reducing serum TC, LDL-C, and HDL-C levels in AS model mice. These studies indicate that dietary supplementation with BCAAs has a beneficial effect on the lipid profile, which can help prevent AS.

AS is a chronic inflammatory disease, and the inflammatory response is involved in the development of AS [8,9]. The AKT/NF-κB signaling pathway is a crucial regulatory pathway of the inflammatory response. AKT performs a crucial function in cellular metabolism and the inflammatory response by regulating the activity of many target proteins [39]. The NF-κB pathway is regulated by phosphorylated AKT. NF-κB is a key transcriptional regulator of most inflammatory genes and is involved in the inflammatory response by coordinating the expression of multiple genes that control inflammation, tissue injury, and immune response. Several studies have shown that the NF-κB signaling pathway plays an important role in AS [40,41,42]. This study showed that dietary supplementation with BCAAs inhibited the activation of AKT and NF-κB.

Inflammatory factors, including IL-6, TNF-α, and IL-1β, are released from the organism when NF-κB is activated [43]. IL-6 is an important upstream inflammatory biomarker that can promote the development of AS by driving vascular smooth muscle cell (VSMC) migration and inducing intracellular cholesterol accumulation [44,45]. TNF-α is an essential cytokine that is primarily released by T cells and activated monocyte macrophages. TNF-α promotes atherosclerosis by facilitating platelet aggregation and enhancing LDL transport in endothelial cells [46]. IL-1β is an inflammatory cytokine that enhances the expression of numerous proinflammatory cytokines, and monoclonal antibodies that target IL-1β may protect against cardiovascular disease [47,48,49]. In addition, the increased IL-1β level was positively correlated with the extent of coronary AS [50]. Additionally, MCP-1, a member of the CC chemokine family, is a strong chemokine for monocytes, controlling the migration and infiltration of monocytes and macrophages. Thus, MCP-1 plays a significant role in the development of AS. It was also demonstrated that MCP-1 could increase macrophage numbers and promote oxidized lipids to aggravate AS in ApoE^−/−^ mice [51]. In the current study, serum MCP-1, IL-6, TNF-α, and IL-1β levels were significantly elevated in ApoE^−/−^ mice fed with a high-fat diet, whereas dietary supplementation with BCAAs dramatically decreased the serum levels of these inflammatory factors. Several previous animal studies have similarly demonstrated that supplementation with Leu dramatically reduced serum levels of inflammatory factors, including IL-6, TNF-α, MCP-1, and leptin in mice [30,52], decreased macrophage infiltration at the aorta, and suppressed systemic inflammatory responses to ameliorate AS [38]. Numerous studies have reported that ICAM-1 and VCAM-1 enhance monocyte and endothelial cell adhesion, which promotes AS [53,54]. Aortic root section staining confirmed that dietary supplementation with BCAAs reduced the inflammatory response at aortic lesions. However, it has also been shown that ingestion of BCAAs enhances human platelet activity and promotes arterial thrombosis in mice [55]. Activated platelets initiate inflammatory responses and participate in AS through multiple pathways. Results that are inconsistent with the above studies may be observed because the effects of dietary supplementation with BCAAs on AS are influenced by the ratio and dose of BCAAs and the approach of administration. Further studies are needed to investigate these differences in the future.

Branched chain amino acid transaminases (BCATs) and particularly Branched chain alpha-ketoacid dehydrogenase (BCKDH) (the rate-limiting enzyme in BCAA catabolism), which are essential for maintaining BCAA homeostasis, regulate BCAA catabolism in vivo. BCKDH-E1α is primarily responsible for BCKDH activity, which is regulated via phosphorylation and dephosphorylation [56]. BCKD kinase-mediated phosphorylation of BCKDH-E1α can inactivate BCKDH, increasing BCAA levels in serum. Monitoring BCAA levels can serve as an indirect indicator of the catabolism of BCAAs, which is mostly based on dietary supplementation and catabolism of BCAAs in vivo. Therefore, serum BCAA levels and liver *p*-BCKDH-E1α and BCKDH-E1α expression levels were examined in this study to analyze BCAA metabolism. Our previous study showed that the enzymatic activity of BCKDH was impaired by HFD feeding and that dietary supplementation with BCAAs boosted the enzymatic activity and counteracted the accumulation of BCAAs in circulation [28]. However, in this study, total serum BCAA levels were significantly increased in the dietary BCAA supplementation group, but no significant differences in the levels of each BCAA or BCKDH-E1α enzyme activity was noted among the four groups, suggesting that the increased serum BCAA levels in ApoE^−/−^ mice may primarily be attributable to dietary BCAAs supplementation. Considering that BCAA homeostasis in vivo is related to the balance between BCAA intake and BCAA metabolism, our studies indicated that BCAA supplementation does not have a dramatic effect on this balance, which is consistent with a previous study in which dietary intake of BCAA was only moderately positively correlated with circulating BCAA levels in humans [57]. Thus, unlike the elevated circulation of BCAA levels being risk factors for AS, BCAA supplementation may have beneficial effects on AS, since BCAA supplementation does not necessarily lead to the impairment of BCAA catabolism and the accumulation of BCAA in circulation and then cause diseases. In the future, whether BCAA can be used as a supplement for disease prevention and treatment in humans needs to be confirmed by randomized clinical trials.

Recently, a growing number of studies have shown that gut microbiota dysbiosis plays an important role in many diseases, including AS. Dysbiosis can promote AS development by enhancing the systemic inflammatory response. Furthermore, during AS progression, some bacteria can promote AS development, whereas others can inhibit AS formation. *GCA-900066225* is associated with abnormal lipid metabolism, and *Prevotella_9*, *Weissella*, *Leuconostoc* and *Photobacterium* are opportunistic bacteria [58,59]. In the present study, a high-fat diet increased the relative abundance of these harmful bacteria. *Alistipes* plays a protective role in cardiovascular disease, and *Ileibacterium* and *Muribaculum* are associated with attenuating the inflammatory response [60,61,62]. *Desulfovibrio*, also known as sulfate-reducing bacteria, metabolizes and produces hydrogen sulfide, which is harmful to the intestinal epithelium and impairs the mucosal barrier [63]. Similar to previous studies, a high-fat diet dramatically increased the relative abundance of *Desulfovibrio.* We found that a high-fat diet reduced the relative abundance of these beneficial bacteria. In summary, a high-fat diet may exacerbate AS by increasing the abundance of harmful bacteria while reducing the abundance of beneficial bacteria.

In addition, dietary supplementation with BCAAs may have protective benefits for AS by reducing the abundance of harmful bacteria and increasing the abundance of probiotics. These effects may be beneficial for intestinal health. Dietary supplementation with BCAAs decreased the relative abundance of *Faecalibaculum* and *Fusobacterium*, two genera of opportunistic pathogenic bacteria that boost inflammatory responses and impair the intestinal barrier. Duan et al. [64] reported that overexpression of *Prevotellaceae_UCG-003* suppresses the inflammatory response, and dietary supplementation with BCAAs significantly increased its relative abundance in the current study. The abundance of *Succinivibrio* in patients with metabolic disorders was much lower than that in healthy patients [65], suggesting that *Succinivibrio* is a potential metabolic probiotic and that dietary supplementation with BCAAs increased its relative abundance.

Cholesterol is converted to BAs to maintain plasma cholesterol levels. Therefore, the upregulation of BA biosynthesis and the excretion of BAs are closely related to low serum cholesterol levels [66]. Cholesterol is the primary factor used in the biosynthesis of BAs, and two main pathways are involved. The classical pathway produces mainly 12α-OH BAs, whereas the alternative pathway produces mainly non-12α-OH BAs. A low proportion of non-12α-OH BAs has been shown to have beneficial metabolic effects [67,68,69]. In addition, primary BAs play critical roles in cholesterol metabolism, lipid digestion, and host-microbe interactions, and can be converted to secondary BAs by the intestinal flora. High-fat diets increase some secondary BAs, which are risk factors for colonic inflammation and cancer [70]. Our results similarly showed that a high-fat diet caused an increase in secondary BAs and a decrease in the proportion of non-12α-OH BAs, indicating that a high-fat diet has a negative effect on ApoE^−/−^ mouse metabolism. In addition, the levels of total BAs, primary BAs, 12α-OH and non-12α-OH BAs in cecal and colonic contents were all increased in the HFB group compared with the HFD group. This finding indicates that dietary supplementation with BCAAs may enhance the excretion of total BAs, mainly increasing primary BAs, which can affect lipid metabolism and exert a metabolic benefit.

Correlation analysis revealed that lipid-related parameters were significantly negatively associated with the relative abundance of *Family_XIII_UCG-001* and slightly negatively associated with the relative abundance of *Ileibacterium* and *Papillibacter*. In contrast, dietary supplementation with BCAAs significantly increased the relative abundance of the three bacteria. In addition, serum lipids showed a significant positive correlation with the relative abundance of *Photobacterium* and a mild positive correlation with the relative abundance of *Lactococcus*. In contrast, dietary supplementation with BCAAs significantly reduced the relative abundance of both. It has been reported that *Bacteroides* is associated with intestinal catabolism of BCAAs, whereas *Streptococcus* is associated with intestinal biosynthesis of BCAAs [71,72]. However, a correlation between *Bacteroides* and *Streptococcus* and serum BCAA levels was not observed in this study, which may be due to the use of different animal models and individual differences in animals. We also found that BA levels were correlated with probiotic bacteria, such as *Bifidobacterium*, *Lactobacillus*, *Phascolarctobacterium*, and *Prevotellaceae_UCG-003*, consistent with previous reports [73,74,75,76]. Overall, dietary supplementation with BCAAs may promote BA metabolism by upregulating the relative abundance of probiotics, such as *Prevotellaceae_UCG-003*.

## 5. Conclusions

In conclusion, this study demonstrates that dietary supplementation with BCAAs may not only alleviate AS by suppressing the inflammatory response, but may also regulate bile acid excretion by altering intestinal flora in ApoE-deficient mice. This research supported the beneficial effects of BCAAs on inflammation and elucidated their potential mechanisms, providing different perspectives on the molecular mechanisms of BCAA supplementation to alleviate AS.

## Data Availability

The data that support the findings of this study are available from the corresponding author upon reasonable request.
